# Should the 14‐day rule for embryo research become the 28‐day rule?

**DOI:** 10.15252/emmm.201809437

**Published:** 2018-08-07

**Authors:** John B Appleby, Annelien L Bredenoord

**Affiliations:** ^1^ Lancaster Medical School Lancaster University Lancaster UK; ^2^ Department of Medical Humanities Julius Center University Medical Center Utrecht Utrecht The Netherlands

**Keywords:** Development & Differentiation, Stem Cells

## Abstract

The “14‐day rule”—broadly construed—is used in science policy and regulation to limit research on human embryos to a maximum period of 14 days after their creation or to the equivalent stage of development that is normally attributed to a 14‐day‐old embryo (Hyun *et al*, 2016; Nuffield Council on Bioethics, 2017). For several decades, the 14‐day rule has been a shining example of how science policy and regulation can be developed with interdisciplinary consensus and applied across a number of countries to help fulfil an ethical and practical purpose: to facilitate efficient and ethical embryo research. However, advances in embryology and biomedical research have led to suggestions that the 14‐day rule is no longer adequate (Deglincerti *et al*, 2016; Shahbazi *et al*, 2016; Hurlbut *et al*, 2017). Therefore, should the 14‐day rule be extended and, if so, where should we draw a new line for permissible embryo research? Here, we provide scientific, regulatory and ethical arguments that the 14‐day rule should be extended to 28 days (or the developmental equivalent stage of a 28‐day‐old embryo).

## Background of the 14‐day rule

In 1978, the birth of Louise Brown, the world's first IVF baby, marked a major clinical breakthrough and demonstrated that it is possible to create and sustain human embryos *in vitro*. These embryos could be used for research or to attempt a pregnancy. In response, the Ethics Advisory Board of the US Department of Health, Education and Welfare held a detailed consultation and published a report in 1979, which cautiously supported human embryo research. However, one of the key conditions that the report proposed was that embryos will not be kept alive *in vitro* longer than 14 days after fertilisation or the stage of development that is equivalent to when embryos finish implantation. At the time, it was still a challenge to keep embryos alive *in vitro* and 14 days seemed like more than enough time to conduct research on them.

In biological terms, the 15^th^ day of embryo development is the point when the primitive streak forms: that is, the beginning of gastrulation when three layers of germ cells differentiate. The 14^th^ day is therefore notable, because the embryo is then individuated and can no longer become a twin. Consequently, the 14^th^ day has, until recently, represented a natural and convenient biological turning point at which to restrict any further research on embryos.

When the UK assembled the Committee of Inquiry Into Human Fertilisation and Embryology in 1982—what became known as the “Warnock Committee”—to debate developments surrounding assisted conception, the idea of a 14‐day limit on embryo research was adopted as part of the Committee's recommendations. These recommendations were published in 1984 in what is now known as the “Warnock Report”. This 14‐day “rule” for embryo research has since formed part of the regulations in the UK's Human Fertilisation and Embryology Act 1990 (as amended) until the present date, and UK embryo research is licensed by the Human Fertilisation and Embryology Authority (HFEA). Since 1979, the 14‐day rule has also been upheld by the US National Institutes of Health's Human Embryo Research Panel. Additionally, it has been implemented, albeit in different ways, by regulators and policymakers around the world, such as in Canada, Australia, India, Japan, the Netherlands and others (Hyun *et al*, 2016); this makes the 14‐day rule one of the most internationally agreed rules in reproductive science and medicine to date.

However, current research in the United States and the UK has demonstrated that it is possible to culture embryos to the equivalent of 13 days and potentially longer (Deglincerti *et al*, 2016; Shahbazi *et al*, 2016). Given that it is now within the technical reach to investigate the developmental nature of embryos beyond 14 days, what is science and society to make of this situation? Should the 14‐day rule be extended?

## Arguments against extending the 14‐day rule

Some have argued that the 14‐day rule was never meant to represent a firm moral boundary for embryo research, but instead a practical time limit (Hyun *et al*, 2016). Yet, many would agree that since its creation, the 14‐day rule has been attributed moral significance for a variety of reasons (Nuffield Council on Bioethics, 2017). As a result, some will argue that any attempt to extend the limit for embryo research to 28 days would be morally problematic. Therefore, before discussing some of the reasons why it would be beneficial to extend the 14‐day rule, we first turn to some of the arguments against extending it. In doing so, we assume that any countries, groups or professional bodies that already employ the 14‐day rule have already adopted a position that permits or is in favour of human embryo research; therefore, we do not focus on discussing general arguments that reject embryo research altogether.

Some might argue that the embryo acquires moral standing after the 14^th^ day, because the onset of gastrulation signifies that the embryo is a distinct individual and therefore has a greater potential for personhood (Hyun *et al*, 2016). However, embryos used for research—as opposed to attempting a pregnancy—are already designated for destruction at 14 days and have no potential for personhood in these circumstances. Therefore, it is unclear in this instance how matters related to potential personhood are ultimately made morally worse by delaying the destruction of embryos for up to 28 days.

Another argument is that conducting research on embryos after the formation of the primitive streak risks the embryo experiencing pain and suffering. In fact, the Warnock Report viewed the 14‐day rule as a way to avoid research being carried out on humans in development who may have some level of sentience (Nuffield Council on Bioethics, 2017). However, at 28 days, no functional neural connections or sensory systems exist in the embryo (Hurlbut *et al*, 2017). It is therefore impossible for the embryo to experience sentience, pain or suffering within this extended period of research. While concerns related to pain and suffering should be taken seriously, it does not appear that they apply to our proposed period of extended embryo research.

Critics of our proposed extension to 28 days may also argue that it would begin a slide down a slippery slope towards an ever‐increasing time window for embryo research. Others may not take issue, in principle, with extending embryo research to 28 days, but may nevertheless be concerned that this could facilitate the further development of technologies that they disagree with, such as germline gene editing (Nuffield Council on Bioethics, 2017). While it would be disappointing if we immediately allowed such objections to trump carefully reasoned, well‐defined and beneficial reforms to embryo research policy, these concerns can however be recognised as broader criticisms of how science policy and regulations are made and enforced in society.

The emergence of slippery slope arguments in discourse surrounding scientific research is often indicative of a broader crisis of confidence and trust in the way science policies, and regulations are debated, crafted and implemented. Given the fact that research and innovation in reproductive biomedicine have historically lacked robust oversight or regulation (both within many countries and internationally), such crises of trust and confidence should be taken seriously and not be dismissed as irrelevant. Therefore, any policy consultation or process of reforms regarding the extension of the 14‐day rule should be open, transparent, informed by evidence and should engage with the broad range of views that surround this topic (Cavaliere, 2017).

## Reasons for extending the 14‐day rule

The period between the 14^th^ and 28^th^ day of embryo development is sometimes referred to as the “black box” of human development (Hurlbut *et al*, 2017; Nuffield Council on Bioethics, 2017). To date, it has been—from a scientific or regulatory point of view—very challenging to study and gain knowledge about how embryos develop during this period. However, a recent Nuffield Council on Bioethics publication on embryo research rightly argues that any attempt to extend the 14‐day rule would need to be based at least on the prospect of important advances in science (Nuffield Council on Bioethics, 2017). Therefore, we outline how scientific progress and biomedical innovation could likely be achieved with the help of extending the 14‐day rule.

Extending the window for embryo research to 28 days would allow scientists to study the developmental processes during gastrulation when the first primitive tissues form. They could learn more about the developing nervous system without any risk of neural connections being present and gain a better understanding of the early development of organs (Hurlbut *et al*, 2017). It would also be possible to advance our knowledge about cell fate decisions during early embryonic development (Shahbazi *et al*, 2016). Moreover, an extended research window could further improve the safety and success rate of current IVF procedures. For example, this could potentially help scientists understand the nature of some birth defects and also help clinicians predict which IVF embryos are likely to result in a successful pregnancy (Hurlbut *et al*, 2017). Scientists could also learn more about the physiology of pregnancy beyond the 14^th^ day, including the processes surrounding implantation and why medical events such as miscarriages happen (Hurlbut *et al*, 2017).

Nevertheless, a number of practical barriers need to be overcome to facilitate the study of embryos beyond 14 days. For instance, scientists must devise and improve ways of keeping an embryo supported and alive in an appropriate environment (Aach *et al*, 2017). This is a difficult challenge, but may be overcome with the help of new technologies, such as 3D bioprinting and organoids (Aach *et al*, 2017; Bredenoord *et al*, 2017). Indeed, extending the 14‐day limit would give researchers the opportunity to learn how to keep embryos alive *in vitro* for longer.

### Benefits to research on stem cell‐derived gametes and gene editing

Scientists have also succeeded in creating stem cell‐derived gametes (SCDGs): egg and sperm cells that have been derived *in vitro* from stem cells. The potential value of SCDGs is immense. They could be used to create an unlimited source of male and female gametes to potentially allow prospective parents with infertility and same‐sex couples to have children that they are genetically related to. As research advances our knowledge and capacity to create SCDGs, it will also become possible to use these to create embryos and study them beyond 14 days. However, before researchers can use SCDGs to create human embryos for assisted reproduction, it would be essential to establish the safety and efficacy of SCDG techniques in order to understand as much as possible about their *in vitro* development—a 28‐day research limit would greatly help with this.

In addition, embryo modification using gene editing has opened up a new frontier of research. In the future, it might be possible to edit genomes in order to treat genetic diseases, such as Huntington's disease. UK researchers have already used CRISPR‐Cas9 to edit the nuclear DNA of a human embryo in order to study how the removal of the *OCT4* gene effects early embryo development (Fogarty *et al*, 2017). While this is groundbreaking research, it would also potentially be helpful to conduct research on CRISPR‐Cas9‐edited embryos beyond the 14^th^ day of development in order to understand the science, efficacy and safety of these radically new technologies (Nuffield Council on Bioethics, 2017).

### Benefit to research on “synthetic” embryos and organoids

Another scientific reason for extending the 14‐day rule is the groundbreaking stem cell research that has made it possible to create so‐called “synthetic” or stem cell‐based embryos (Aach *et al*, 2017) and organoids. Human pluripotent stem cells can be cultivated *in vitro* to derive‐self organising cell structures with features that resemble early human development (Warmflash *et al*, 2014; Aach *et al*, 2017). These structures have been referred to as gastruloids (Aach *et al*, 2017) or synthetic human entities with embryo‐like features (SHEEFs; Aach *et al*, 2017). While SHEEFs are not intact embryos and are not totipotent, it may be possible to eventually create totipotent “synthetic embryos” (Warmflash *et al*, 2014; Deglincerti *et al*, 2016; Shahbazi *et al*, 2016; Aach *et al*, 2017). The main difference is that synthetic embryos would be derived from human pluripotent stem cells, as opposed to an egg and sperm. Synthetic embryos could be valuable for creating a limitless supply of research embryos, which of course poses ethical questions in itself, and potentially for creating embryos for infertile persons who wish to have children without the need to use sperm or egg donors. Again, it will be valuable for establishing safety and efficacy, to be able to study these embryos beyond 14 days up until the 28^th^ day of development.

Extending the 14‐day rule on embryo research would also benefit research on organoids, which are three‐dimensional structures that are grown *in vitro* using stem and progenitor cells (Bredenoord *et al*, 2017). These miniature models of organ tissue can form eye, brain, kidney or intestinal tissues (among other forms) and are valuable and versatile research tools in biomedicine. For example, they can be used in their own right to understand the physiology and development of organs or as personalised and precise human models for drug testing. In addition, organoids of human tissue are often more safe, efficient and accurate than animal research models, which can help to reduce the use of animals in biomedical research. However, for the sake of safety and accuracy, the results derived from human organoids must be corroborated with other research models (Bredenoord *et al*, 2017). By extending the 14‐day rule, human embryos could be cultured *in vitro* to act as effective models for testing and verifying organoid research findings, as embryos begin to develop specialised cells and tissue precursors after 14 days.

Moreover, extending the 14‐day rule could create opportunities to integrate embryo research with organoid research. Organoids can potentially be used to model the specific tissue niches that embryos use for implantation and development during pregnancy. They could therefore be used as a “natural” 3D support structure for the development and implantation of embryos, but also as dynamic biological models to help scientists understand what makes pregnancies successful and what causes miscarriages (Bredenoord *et al*, 2017). Only extending the 14‐day rule will allow researchers to combine organoid and embryo research to gain this level of in‐depth insight into the early stages of human pregnancy.

## Ethics, policy and governance

Although the 14‐day rule is viewed by many as a success, it must be “fit for purpose” to remain effective and relevant; it should not become a dogma in itself. Science is changing and regulations need to adapt. There is insufficient global governance of ARTs, but the 14‐day rule is one example of how governance is widely adopted (albeit with varying interpretations) and works well. However, a failure to revise the 14‐day rule places the international community at risk of losing one of its better examples of international consensus and regulation, because the rule itself could be viewed as no longer fit for purpose. Unwillingness by policymakers to reconsider and revise the 14‐day rule would send a damaging message to both medical innovators and those in society—notably, patients—who stand to benefit from research. Novel reproductive research will require revised or new regulation (for instance, better regulation of mitochondrial replacement techniques, gene editing techniques for embryos and SCDGs), including an extension of the 14‐day rule, in order to transition from “bench to bedside” safely and responsibly.

Should embryo research between day 14 and day 28 be treated with more regulatory scrutiny than research during the first 14 days? Of course, the answer depends on the country and its regulations. Some countries already have a rigorous regulatory framework. For example, the UK has the Human Fertilisation and Embryology Act 1990 (as amended) and a regulator (the Human Fertilisation and Embryology Authority—the HFEA) for licensing embryo research within the first 14 days. Currently, the HFEA will only permit research with a clear purpose and it must be licensed. Such a model could be extended to the end of the 28^th^ day in order to ensure rigour and consistency.

Any consultation model for amending the 14‐day rule should involve discussion with the public and a multidisciplinary array of experts, in the form of what we have called a modern “Reproductive Asilomar”. For example, when implementing and reviewing the 14‐day rule, both the United States and the UK have historically created forums to share moral views and scientific preferences. The inclusive nature of such regulatory consultations has undoubtedly been responsible for stronger trust in the 14‐day rule and the process that led to its implementation. Any new consultations should therefore aim to promote inclusivity and trust.

We have argued that there are good reasons for extending the 14‐day rule to 28 days. Allowing scientists to conduct research on embryos could benefit science and patients. Furthermore, in countries that already permit embryo research until 14 days, it is difficult to identify any compelling moral arguments against extending this limit to 28 days. In order for embryo research to fulfil its potential benefit to humans both now and in the future, we therefore propose that the current limit on research should be extended to 28 days or the equivalent developmental stage that is normally attributed to a 28‐day‐old embryo.

**BOX: Further reading**

*Ad Hoc* Group of Consultants to the Advisory Committee to the Director, NIH (1994) *Report of the Human Embryo Research Panel*. Washington, DC: US Government Printing Office

Appleby JB (2015) The ethical challenges of the clinical introduction of mitochondrial replacement techniques. *Med Health Care Philos* 18: 501–514

Bredenoord AL, Appleby JB (2017) Mitochondrial replacement techniques: remaining ethical challenges. *Cell Stem Cell* 21: 301–304

Bredenoord AL, Hyun I (2017) Ethics of stem cell‐derived gametes made in a dish: fertility for everyone? *EMBO Mol Med* 9: 396–398

Chan S (2017) How to rethink the fourteen‐day rule. *Hastings Cent Rep* 47: 5–6

Cyranoski D (2016) Mouse eggs made from skin cells in a dish. *Nature* 538: 301

Denker HW (2014) Stem cell terminology and ‘synthetic’ embryos: a new debate on totipotency, omnipotency, and pluripotency and how it relates to recent experimental data. *Cells Tissues Organs* 199: 221–227

Dondorp W, de Wert G (2011) Innovative reproductive technologies: Risks and responsibilities. *Hum Reprod* 26: 1604–1608

Ethics Advisory Board, Department of Health, Education, and Welfare (1979) *HEW Support of Research Involving Human In Vitro Fertilization and Embryo Transfer*. Washington, DC: US Government Printing Office

Harper J, Magli MC, Lundin K, Barratt CLR, Brison D (2012) When and how should new technology be introduced into the IVF laboratory. *Hum Reprod* 27: 303–313

Herrick JR, Paik T, Strauss KJ, Schoolcraft WB, Krisher RL (2016) Building a better mouse embryo assay: effects of mouse strain and *in vitro* maturation on sensitivity to contaminants of the culture environment. *J Assist Reprod Genet* 33: 237–245

Hikabe O, Hamazaki N, Nagamatsu G, Obata Y, Hirao Y, Hamada N, Shimamoto S, Imamura T, Nakashima K, Saitou M *et al* (2016) Reconstitution *in vitro* of the entire cycle of the mouse female germ line. *Nature* 539: 299–303

Itskovitz‐Eldor J, Schuldiner M, Karsenti D, Eden A, Yanuka O, Amit M, Soreq H, Benvenisty N (2000) Differentiation of human embryonic stem cells into embryoid bodies compromising the three embryonic germ layers. *Mol Med* 6: 88–95

Jasanoff S, Hurlbut JB (2018) A global observatory for gene editing. *Nature* 555: 435–437

Menezo Y, Dale B, Elder K (2017) Time to re‐evaluate ART protocols in the light of advances in knowledge about methylation and epigenetics: an opinion paper. *Hum Fertil (Camb)* 21: 156–162

Pera MF, de Wert G, Dondorp W, Lovell‐Badge R, Mummery CL, Munsie M, Tam PP (2015) What if stem cells turn into embryos in a dish? *Nat Methods* 12: 917–919

UK Department of Health and Social Security (1984) *Report of the Committee of Inquiry into Human Fertilisation and Embryology*. London, UK: Her Majesty's Stationary Office

US National Academies of Sciences, Engineering, and Medicine (2017) *Human genome editing: science, ethics, and governance*. Washington, DC: The National Academies Press

van den Brink SC, Baillie‐Johnson P, Balayo T, Hadjantonakis AK, Nowotschin S, DA Turner DA, Martinez Arias A (2014) Symmetry breaking, germ layer specification and axial organisation in aggregates of mouse embryonic stem cells. *Development* 141: 4231–4242

Zhou Q, Wang M, Yuan Y, Wang X, Fu R, Wan H, Xie M, Liu M, Guo X, Zheng Y *et al* (2016) Complete meiosis from embryonic stem cell derived germ cells *in vitro*. *Cell Stem Cell* 18: 330–340
